# Sjögren's syndrome manifesting as clinicopathological features of TAFRO syndrome: A case report

**DOI:** 10.1097/MD.0000000000009220

**Published:** 2017-12-15

**Authors:** Shino Fujimoto, Hiroshi Kawabata, Nozomu Kurose, Haruka Kawanami-Iwao, Tomoyuki Sakai, Takafumi Kawanami, Yoshimasa Fujita, Toshihiro Fukushima, Yasufumi Masaki

**Affiliations:** a Department of Hematology and Immunology, Kanazawa Medical University; b Department of Pathology and Laboratory Medicine, Kanazawa Medical University, Daigaku, Uchinada, Ishikawa-ken, Japan.

**Keywords:** Castleman disease, Sjögren's syndrome, TAFRO syndrome

## Abstract

**Rationale:**

TAFRO syndrome is a newly proposed disorder that manifests as thrombocytopenia, anasarca, fever, reticulin myelofibrosis, renal dysfunction, and organomegaly. In this report, we describe the development of severe TAFRO syndrome-like systemic symptoms during the clinical course of juvenile-onset Sjögren's syndrome in a 32-year-old woman.

**Patient concerns:**

The patient was admitted due to dyspnea, fever, polyarthralgia, and generalized edema. She had been diagnosed with Sjögren's syndrome at the age of 14 years, based on histopathological examination of a biopsy of the minor salivary glands and the development of Raynaud's phenomenon, with no follow-up treatment required. On admission, she presented with anemia, elevated C-reactive protein levels, anasarca, and hepato-splenomegaly. A bone marrow examination revealed increased megakaryocytes with reticulin fibrosis, and the histopathology of an axillary lymph node was consistent with mixed-type Castleman disease. Eventually, she developed thrombocytopenia.

**Interventions:**

Her symptoms fulfilled all of the major and minor categories of the diagnostic criteria for TAFRO syndrome. However, considering her prior diagnosis, we assumed that the clinical presentation was consistent with an acute exacerbation of Sjögren's syndrome. Unlike typical cases of TAFRO syndrome, the administration of relatively low-dose prednisolone relieved her symptoms.

**Lessons:**

Differentiation between TAFRO syndrome and exacerbation of an autoimmune disease is clinically important, although this can be challenging. Identification of specific biomarkers for TAFRO syndrome would be clinically beneficial.

## 1 Introduction

TAFRO syndrome is a newly proposed disorder that manifests as thrombocytopenia, anasarca (pleural effusion/ascites and systemic edema), fever, reticulin myelofibrosis, renal dysfunction, and organomegaly (hepatosplenomegaly and lymph node swelling).^[1,2]^ In most cases, the onset of this syndrome is acute or subacute, and leads to rapid deterioration in health status. According to the diagnostic criteria that were proposed for TAFRO syndrome in 2015,^[3]^ all 3 major categories, that is, anasarca (pleural effusion, ascites, and/or generalized edema), thrombocytopenia, and inflammatory signs/symptoms should be met; and at least 2 of the 4 minor categories, that is, Castleman disease-like lymph node histopathology, reticulin myelofibrosis or increased megakaryocytes in the bone marrow, organomegaly, and progressive renal insufficiency, should be met for diagnosis.^[3]^ In addition, malignancies, autoimmune diseases, infectious diseases, POEMS syndrome, IgG4-related disease, hepatic cirrhosis, and thrombotic microangiopathies should be excluded. In fact, patients with autoimmune diseases, such as systemic lupus erythematosus (SLE) and vasculitis syndrome, sometimes show systemic inflammatory symptoms similar to those of TAFRO syndrome,^[4]^ probably due to an overproduction of inflammatory cytokines. In this report, we describe the case of a patient who presented with severe TAFRO syndrome-like systemic symptoms, which developed during the clinical course of juvenile-onset Sjögren's syndrome, a common autoimmune disease.

## 2 Consent

A written informed consent was obtained from the patient for the publication of this case report.

## 3 Case presentation

### 3.1 Patient information

A 32-year-old woman was admitted to Kanazawa Medical University Hospital due to dyspnea, fever, polyarthralgia, and generalized edema. At the age of 14, she was diagnosed with Sjögren's syndrome, based on histopathological examination of a biopsy of the minor salivary glands. She had a history of Raynaud's phenomenon, stiffness of the hand joints, and subclinical levels of sicca symptoms, which did not require any specific treatment.

### 3.2 Clinical findings and diagnostic assessment

**Figure 1:**
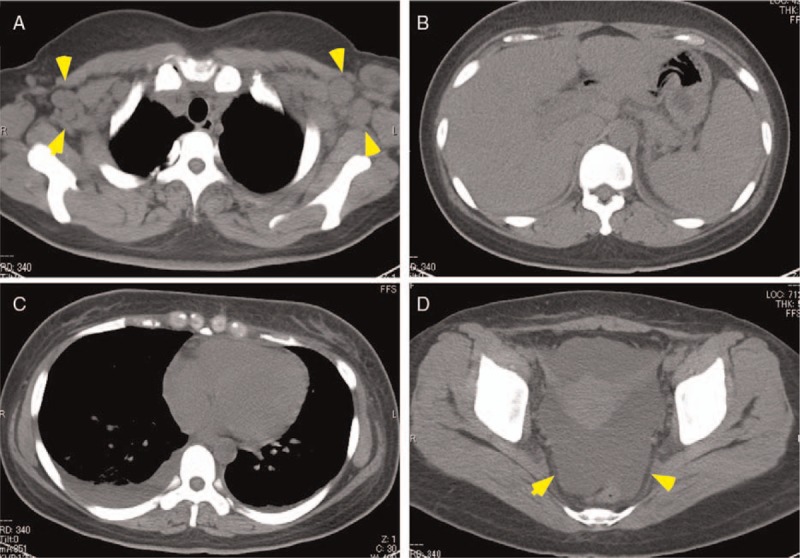
Computed tomography images obtained on admission, showing: (A) bilateral axillary lymphadenopathy (arrow heads), (B) bilateral pleural effusion, (C) hepatosplenomegaly, and (D) massive ascites (arrow heads).

**Figure 2:**
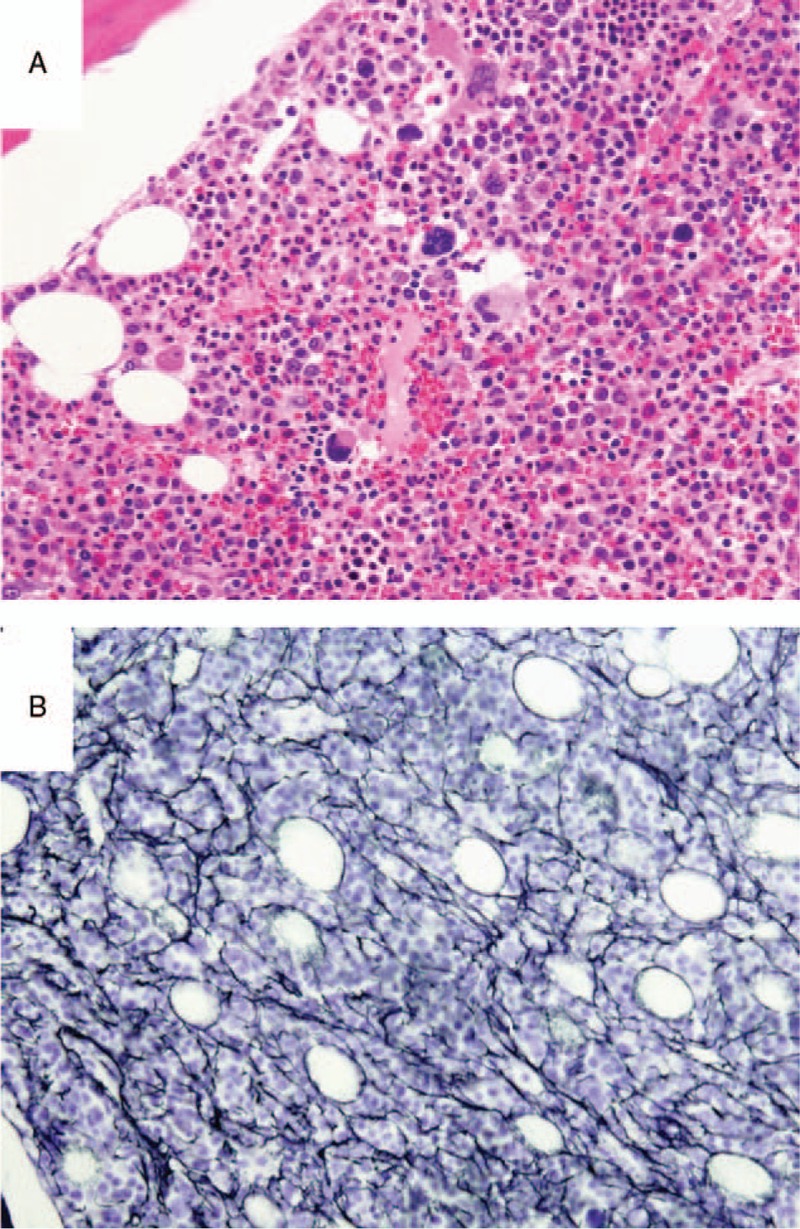
Sections of the bone marrow biopsy, showing: (A) a slightly hypercellular marrow with increased megakaryocytes was observed (hematoxylin and eosin staining, ×100) and (B) reticulin fibrosis (MF-2; reticulin staining, ×100).

**Figure 3:**
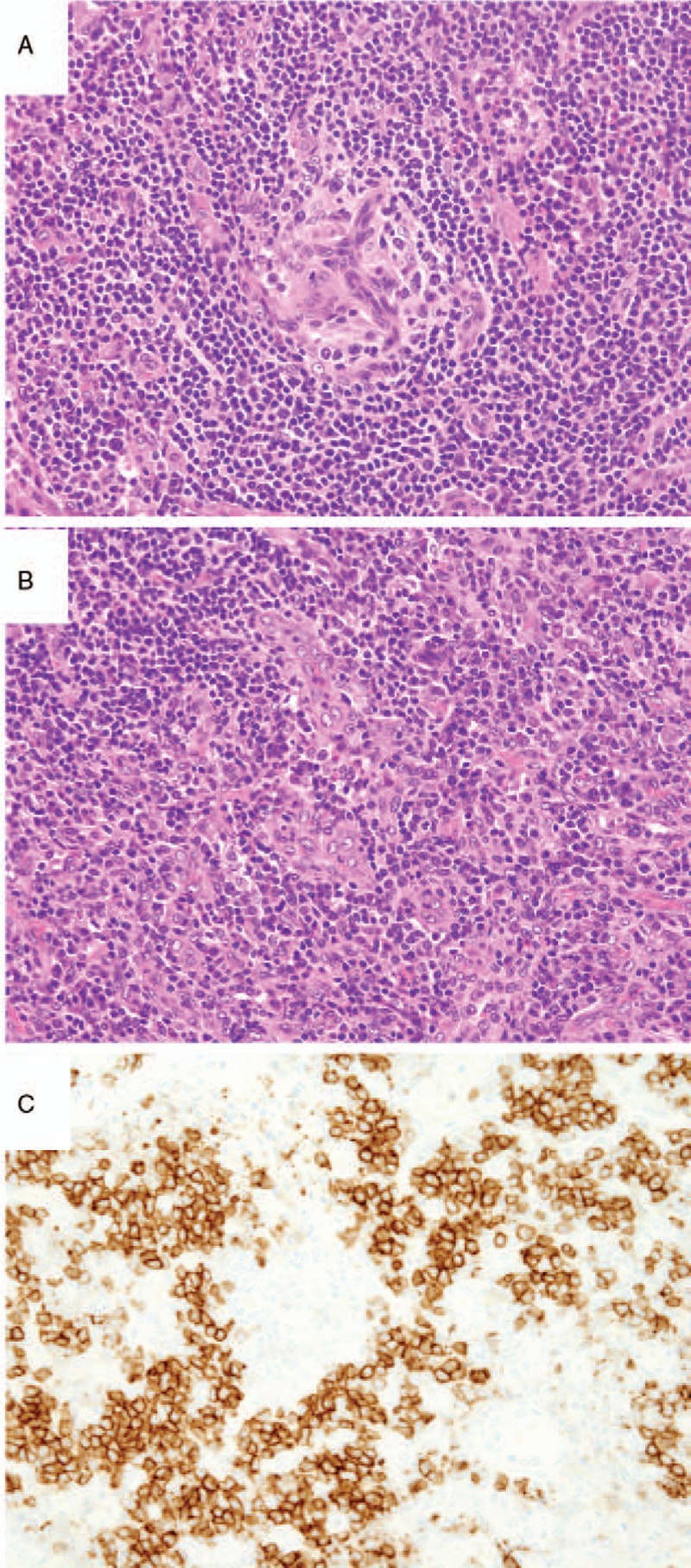
Sections of a left axillary lymph node. The architecture of the lymph node was conserved with relatively small follicles. (A) Proliferation of glomerulus-like blood vessels in the germinal center. (B) Proliferation of endothelial venules and infiltration of plasma cells in the interfollicular spaces. The sections that appear in panels (A) and (B) were stained with hematoxylin and eosin. (C) Infiltration of CD38-positive plasma cells into the interfollicular spaces, but not inside the follicles (immunohistochemical staining using an anti-CD38 antibody). Original magnification, ×100.

**Figure 4:**
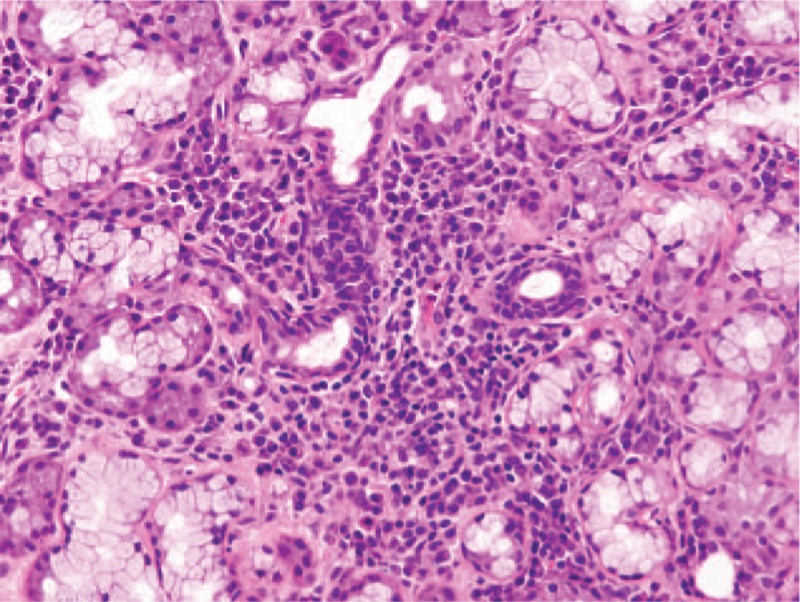
A section of the minor salivary gland biopsy (hematoxylin and eosin staining; original magnification, ×100), showing infiltration of lymphocytes and plasma cells in the periductal regions, lymphocytic infiltration into the ductal epithelium, and nuclear pseudostratification of the ductal epithelium (Grade 4 sialoadenitis, with a focus score of 6.5).

On admission, her body temperature was 38.2°C, her blood pressure 118/64 mm Hg, and her pulse rate 82 beats/min. Dryness of the oral mucosa and tongue were observed. Her heart and respiratory sounds were normal. Her liver was palpable, 3.75 cm below the right costal margin, and the spleen was palpable, 1.5 cm below the left costal margin. Elastic hard lymph nodes were palpable in the left (diameter, 2.5 cm) and right (diameter, 1.5 cm) axillary regions. Marked pitting edema of the lower limbs was observed. Laboratory data revealed normocytic anemia (hemoglobin level, 8.5 g/dL), borderline thrombocytopenia (128 × 10^3^ /μL) and an elevated C-reactive protein (CRP) level (7.45 mg/dL). Her serum anti-nuclear antibody test was positive (160-folds, speckled pattern), and her anti-SS-A and -SS-B antibody tests were also positive (Table 1). Urinalysis showed proteinuria (1.85 g/day), a urinary protein selectivity index (IgG to transferrin) of 0.07, elevated β2-microgloburin (β2MG) level (2533 μg/L, reference range, <230 μg/L), and elevated *N*-acetyl-β-D-glucosaminidase (NAG) level (15.3 IU/L, reference range, 0.7–11.2 IU/L). Very few red blood cells and a few granular and epithelial casts were identified in her urine. A computed tomography scan revealed bilateral axillary lymphadenopathy, small amounts of bilateral pleural effusion, hepato-splenomegaly, and massive ascites (Fig. 1). A bone marrow examination revealed an increase in megakaryocytes with reticulin fibrosis (MF-2) (Fig. 2). A left axillary lymph node biopsy revealed proliferation of endothelial venules and infiltration of CD38-positive plasma cells in the interfollicular area, similar to the findings of mixed-type Castleman disease (Fig. 3). The prior diagnosis of Sjögren's syndrome was confirmed by histopathological findings of the minor salivary glands (Fig. 4), salivary gland scintigraphy (data not shown) and ophthalmological examination. After admission, the patient was treated with furosemide and celecoxib, which resolved her fever and gradually decreased her CRP level. However, her dyspnea, proteinuria and generalized edema did not improve, and the platelet count gradually decreased to 64 × 10^3^ /μL at 4 weeks after treatment initiation. Thus, her symptoms fulfilled all 3 major categories (thrombocytopenia, anasarca, and systemic inflammation) and all 4 minor categories (Castleman disease-like lymph node histopathology, reticulin myelofibrosis/increased megakaryocytes in the bone marrow, organomegaly, and renal dysfunction) of TAFRO syndrome. However, because she had an underlining autoimmune disease, we considered that her symptoms were due to an exacerbation of Sjögren's syndrome.

**Table 1:**
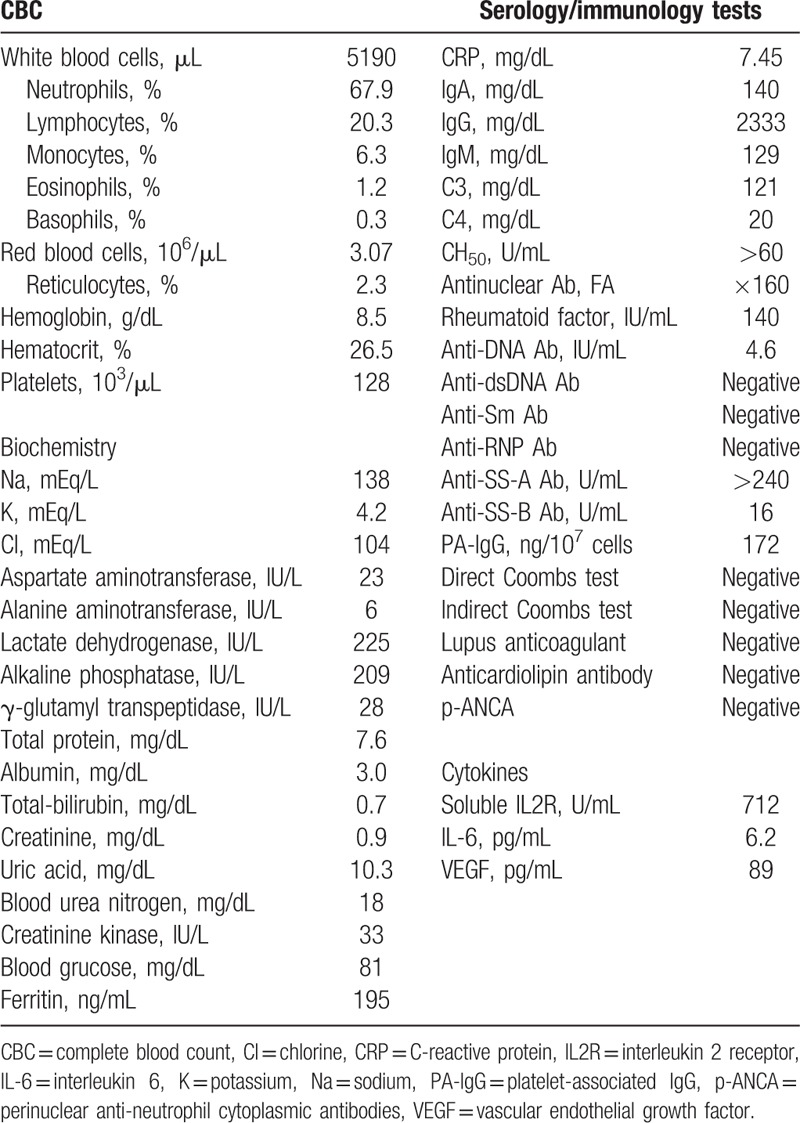
Blood test results on admission.

### 3.3 Therapeutic interventions

**Figure 5:**
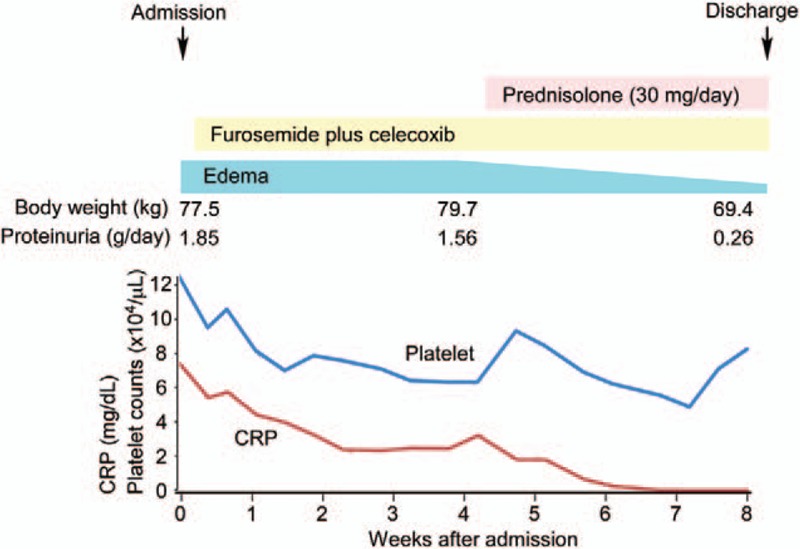
The patient's clinical course, with the time line of treatments and symptoms, as well as platelet counts (blue line) and serum CRP levels (red line). CRP = C-reactive protein.

Prednisolone (30 mg/day) gradually resolved the TAFRO syndrome-like symptoms, including fever, thrombocytopenia, anasarca, and organomegaly (Fig. 5). The patient was discharged one month after prednisolone was initiated.

## 4 Discussion

Sjögren's syndrome is an autoimmune disease that is characterized by dry eyes and mouth (sicca symptoms) due to lymphocytic infiltration of the lacrimal and salivary glands. Most patients are female and the disease is commonly diagnosed in middle-aged and elderly individuals. In the current case, however, Sjögren's syndrome was diagnosed in early adolescence, when the patient had Raynaud's phenomenon with subclinical sicca symptoms. Because patients with mild sicca symptoms may not seek medical attention, and that the symptoms generally progress slowly, some “adult-onset” patients with Sjögren's syndrome might have had subclinical sicca symptoms during their childhood that were overlooked.^[5]^

TAFRO syndrome is a systemic inflammatory disorder of unknown etiology that manifests as thrombocytopenia, anasarca, reticulin myelofibrosis, and progressive renal dysfunction.^[1,2,6,7]^ Because the lymph nodes of patients with TAFRO syndrome usually show mixed-type Castleman disease-like histopathological features, and some clinical features of TAFRO syndrome, such as microcytic anemia and elevation of the serum CRP level, overlap with those of idiopathic multicentric Castleman disease (iMCD),^[7]^ some researchers have assumed that TAFRO syndrome is a subtype of iMCD.^[8,9]^ However, in patients with TAFRO syndrome, lymphadenopathy is usually modest or sometimes absent, and some of the clinical characteristics of TAFRO syndrome are clearly different from those of typical iMCD. For example, the onset of TAFRO syndrome is acute or subacute while the onset of iMCD is chronic. The former disorder manifests as thrombocytopenia, while the latter frequently manifests as thrombocytosis. Furthermore, normal serum IgG levels appear in patients with the former disorder, while marked elevation of these levels is present in the latter.

In order to clarify the clinical features of TAFRO syndrome and establish an optimal treatment strategy, we have been conducting a multicenter retrospective study since October 2013 (UMIN000011809), with more than 200 patients with TAFRO syndrome, iMCD, or diseases with similar clinical features having been registered in this database to date. During this study, we have identified cases in which an acute exacerbation of collagen diseases manifests as TAFRO syndrome-like symptoms. In our case, the clinical manifestation met all 3 major and all 4 minor categories of the diagnostic criteria for TAFRO syndrome.^[3]^ Because the patient had been diagnosed with Sjögren's syndrome, we assumed that her TAFRO syndrome-like symptoms were associated with an acute exacerbation of collagen disease. If the patient had not been diagnosed with Sjögren's syndrome, based on a minor salivary gland biopsy, we could have diagnosed her with “idiopathic” TAFRO syndrome, based on her symptoms.

Autoimmune diseases, including Sjögren's syndrome, often manifest lymphadenopathy,^[10]^ and the histopathology of the affected lymph nodes, referred to as atypical lymphoplasmacytic and immunoblastic proliferation,^[11]^ is quite similar to that of iMCD.^[12,13]^ Kojima et al^[6]^ described 7 cases diagnosed with multicentric Castleman disease showing effusion at the initial clinical presentation, with 2 of them were diagnosed as having Sjögren's syndrome over the clinical courses of their disease. Patients with Sjögren's syndrome can also manifest immune thrombocytopenia. A literature review by Liu et al. demonstrated that primary Sjögren's syndrome was the second most common autoimmune disease associated with secondary immune thrombocytopenia.^[14]^ Autoimmune myelofibrosis and pleuritis are relatively common in SLE but not in primary Sjögren's syndrome.^[15,16]^ However, a couple of cases of Sjögren's syndrome with secondary myelofibrosis and several cases with pleuritis have been reported, mainly from East Asian countries.^[17–21]^ A Chinese study in 573 patients with primary Sjögren's syndrome reported an incidence rate of pleural effusion of 5.7%.^[22]^ Ethnic background and/or environmental factors may contribute to the high incidence of pleuritis in these countries. Inflammatory cytokines, which induce proliferation of megakaryocytes, such as platelet-derived growth factor and transforming growth factor-β, may be involved in autoimmune myelofibrosis,^[23]^ with interleukin 6 and vascular endothelial growth factor possibly involved with anasarca and pleuritis. In the current case, it is possible that hypoalbuminemia likely exacerbated the anasarca.

The pathophysiology of renal insufficiency in our case is also unclear. Primary Sjögren's syndrome is often accompanied by tubulointerstitial nephritis, which can cause AA amyloidosis over a prolonged period.^[24]^ Membranous nephropathy, membrano-proliferative glomerulonephritis, focal segmental glomerulosclerosis, and renal vein thrombosis also have been reported in patients with Sjögren's syndrome as causes of proteinuria.^[25,26]^ In our case, we did not perform a renal biopsy due to the progressive thrombocytopenia. However, taking into account the increased levels of urine β2MG and NAG, which are markers of tubular damage, absence of hematuria, the high glomerular size-selectivity and the good response to a relatively low-dose steroid therapy, we assumed that renal insufficiency in our patient was caused by a tubulointerstitial nephritis and minimal change disease with thin basement membrane nephropathy.^[27]^

The treatment strategy for TAFRO syndrome is undetermined. Corticosteroids have been used as the initial treatment for most reported cases of TAFRO syndrome, with this treatment alone being insufficient in most cases to ameliorate the symptoms of TAFRO syndrome. In cases of treatment failure, the addition of immunosuppressive agents, such as cyclosporine A, tocilizumab, or rituximab, has been used to control symptoms.^[28–36]^ In contrast to these previously reported cases, our patient responded well to a relatively low-dose corticosteroid therapy, which seems to be atypical for patients with TAFRO syndrome. Recently, Iwanaga et al^[37]^ reported the case of a 25 year-old Japanese woman with symptoms of Sjögren's syndrome that manifested as TAFRO syndrome-like symptoms. Her symptoms were resistant to corticosteroid therapy and additional treatment with cyclosporine A was required for symptom amelioration. In cases of acute exacerbation of Sjögren's syndrome, presenting with TAFRO syndrome-like features, responses to corticosteroids may vary from case to case.

The etiology of TAFRO syndrome is unknown. Depending on each case, various factors, such as genetic susceptibility, possible viral infections, and paraneoplastic or autoimmune mechanisms may be involved. Because this syndrome has common manifestations among patients, a common factor may cause these manifestations. Recently, Iwaki et al^[38]^ reported that serum interferon γ-induced protein 10 kDa (IP-10) was significantly increased in patients with TAFRO syndrome but not in patients with iMCD without TAFRO syndrome-like symptoms. Although further validation studies are needed, IP-10 may be a useful biomarker for diagnosing TAFRO syndrome.

In summary, we describe the clinical presentation of a patient that resembled TAFRO syndrome, which included anasarca and systemic inflammatory symptoms during the course of juvenile-onset Sjögren's syndrome. The presenting clinical features included lymph nodes with iMCD-like features and TAFRO syndrome-like systemic symptoms, which can develop during the acute exacerbation of collagen diseases including Sjögren's syndrome. Our case indicates that there are clinicopathological links between Sjögren's syndrome, iMCD and TAFRO syndrome, and underscores the difficulty in strictly differentiating these clinical entities in some cases.^[25]^ Identification of specific biomarkers for TAFRO syndrome would be clinically beneficial.
